# Clinical Neuroprotective Drugs for Treatment and Prevention of Stroke

**DOI:** 10.3390/ijms13067739

**Published:** 2012-06-21

**Authors:** Kiyoshi Kikuchi, Hisaaki Uchikado, Motohiro Morioka, Yoshinaka Murai, Eiichiro Tanaka

**Affiliations:** 1 Department of Physiology, Kurume University School of Medicine, 67 Asahi-machi, Kurume 830-0011, Japan; E-Mails: kikuchi_kiyoshi@kurume-u.ac.jp (K.K.); ymurai@med.kurume-u.ac.jp (Y.M.); 2 Department of Neurosurgery, Kurume University School of Medicine, 67 Asahi-machi, Kurume 830-0011, Japan; E-Mails: uchikado@med.kurume-u.ac.jp (H.U.); mmorioka@med.kurume-u.ac.jp (M.M.); 3 Uchikado Neuro-Spine Clinic, 1-2-3 Naka, Hakata-ku, Fukuoka 812-0893, Japan

**Keywords:** stroke, neuroprotective effect, clinical drugs

## Abstract

Stroke is an enormous public health problem with an imperative need for more effective therapies. In therapies for ischemic stroke, tissue plasminogen activators, antiplatelet agents and anticoagulants are used mainly for their antithrombotic effects. However, free radical scavengers, minocycline and growth factors have shown neuroprotective effects in the treatment of stroke, while antihypertensive drugs, lipid-lowering drugs and hypoglycemic drugs have shown beneficial effects for the prevention of stroke. In the present review, we evaluate the treatment and prevention of stroke in light of clinical studies and discuss new anti-stroke effects other than the main effects of drugs, focusing on optimal pharmacotherapy.

## 1. Introduction

Stroke is a major cause of morbidity and disability in industrialized countries and the overall costs of stroke care will account for 6.2% of the total burden of illness by 2020 [1]. The neuronal cell death associated with cerebral ischemia is due to the lack of oxygen and glucose, and may involve the loss of ATP, excitotoxicity of glutamate, oxidative stress, reduced neurotrophic support, and multiple other metabolic stresses [2]. A drug directed against a single molecular target may not be effective at treating the neuronal cell death associated with stroke [3]. Therefore, there is an imperative need for effective preventive therapy, and for early critical care in patients with stroke. Antithrombotic therapies, using tissue plasminogen activators (tPAs), antiplatelet agents and anticoagulants, are currently the most effective type of therapy for ischemic stroke. However, antithrombotic therapy alone is not enough. Drug therapy using broadly neuroprotective drugs may be required for the treatment of stroke during antithrombotic therapy [3]. Regrettably, numerous neuroprotective drugs have failed to demonstrate beneficial effects in Phase II/III clinical trials, despite previous encouraging preclinical results [4]. However, some drugs that have been approved for use in the clinic have neuroprotective effects, and these could be used for the treatment and prevention of stroke in patients.

## 2. Treatment for Stroke

### 2.1. Free Radical Scavengers

A recent review estimated that more than 1000 compounds have been tested in animal models of ischemic stroke; however, none of the 114 compounds that have entered clinical trials have so far proven successful [5]. Several free radical scavengers have been assessed for their efficacy in the treatment of ischemic stroke, but few of these have shown success in studies conducted in Western countries [6]. In contrast, trials conducted in Japan have shown greater levels of success with such scavengers [6].

#### Edaravone

The free radical scavenger edaravone (3-methyl-1-phenyl-2-pyrazolin-5-one, MCI-186, Radicut; Mitsubishi Tanabe Pharma Corporation, Osaka, Japan) exerts antioxidant effects by inhibiting hydroxyl radical-dependent and -independent lipid peroxidation [7,8]. This antioxidant activity, the main proposed mechanism of action, may protect against free radical-related injuries following ischemic stroke [9]. Edaravone also suppresses the increase in the levels of hydroxyl and superoxide anion radicals in several models of ischemic stroke [10,11]. Unlike other free radical scavengers, edaravone readily crosses the blood–brain barrier (BBB) [6], possibly explaining its efficacy where other free radical scavengers have failed to show any. Several lines of evidence have shown that edaravone has neuroprotective effects following brain injury after ischemic stroke. For example, ischemic stroke is associated with enhanced expression of aquaporin 4 (AQP4), which causes acute edema, and the release of high-mobility group box 1 (HMGB1) from affected tissue, worsening neurological outcomes [12,13]. Furthermore, edaravone was found to attenuate ischemic injury by suppressing AQP4 in a rat ischemic stroke model [13]. Moreover, edaravone was found to rescue rats from ischemic stroke by attenuating the release of HMGB1 from the nucleus of neuronal cells in a rat ischemic stroke model [12]. Taken together, these findings suggest that edaravone may be used to treat patients with cerebral infarction by targeting and inhibiting the deleterious molecular events associated with brain injury. Furthermore, it has been reported that edaravone has diverse effects in many organs of several animal diseases models beyond ischemic stroke [14–17].

Edaravone was introduced as the first free radical scavenger for the treatment of stroke [18]. The Japanese Guidelines for the management of stroke 2009 suggest edaravone for the treatment of acute ischemic stroke as a grade B recommendation. Therefore, edaravone is now widely used to treat acute ischemic stroke in Japan [19]. It is currently only approved in Japan. Clinical trial data show that administration of edaravone within 72 h of ischemic stroke onset significantly reduces infarct volume and provides sustained benefits over a 3-month follow-up period [20]. Administration of edaravone within 24 h of ischemic stroke onset has also been performed in patients with lacunae, large-artery atherosclerosis, and cardioembolic stroke, showing beneficial effects on rehabilitation [21,22].

In a retrospective study of acute ischemic stroke patients, Unno *et al.* reported that the total dose of edaravone was associated with gains in rehabilitation [22]. Rehabilitation gain was defined as the change (increase) in the Functional Independence Measure-Motor (FIM-M) or Barthel Index (BI) score from the time of convalescent rehabilitation hospital admission to discharge. Significant correlations were found between the total amount of edaravone used and the degree of increase in FIM-M and BI scores in patients with cardioembolic stroke. Cardioembolic stroke patients also showed improvements in both FIM-M and BI scores as the total amount of edaravone administered increased. Patients with atherothrombotic stroke showed a similar tendency with respect to the change in BI score.

In a randomized, controlled pilot study of acute ischemic stroke patients, long-term treatment with edaravone suppressed the progression of disuse muscle atrophy and improved leg locomotor function to a greater extent than did short-term treatment [21]. Disuse muscle atrophy of the paretic and non-paretic legs was significantly less severe in the long-term treatment group than in the short-term treatment group 3 months after stroke onset. Additionally, maximum walking speed over a distance of 10 m was significantly faster in the long-term treatment group than in the short-term treatment group.

Shinohara and coworkers studied the effects of edaravone in a mixed population of 401 acute ischemic stroke patients, including patients with thrombotic and lacunar stroke [23]. The study was part of a multicenter, randomized, parallel-group, open-label design study comparing edaravone with sodium ozagrel (ozagrel), an antiplatelet agent restricted to use in the treatment of acute noncardioembolic ischemic stroke. Edaravone and ozagrel are used together in many cases in Japan. However, in the study by Shinohara *et al*., these drugs were compared for the first time in Japan. The percentages of patients scoring grade 0–1 on the modified Rankin Scale (mRS) at 3 months were 57.1% and 50.3% in the edaravone and ozagrel groups, respectively. There were no particular concerns over the safety of edaravone. The main conclusion was that edaravone was at least as effective as ozagrel in treating acute noncardioembolic ischemic stroke.

Furthermore, a recent study showed that administration of edaravone during tPA infusion could enhance recanalization in 40 patients with acute ischemic stroke [19]. Of the 40 patients enrolled, 23 patients were assigned to the edaravone group (in which tPA was intravenously infused and intravenous edaravone was started at the same time) and 17 patients were assigned to the non-edaravone group (edaravone was given after the end of tPA infusion). Early recanalization was more frequently observed in the edaravone group (56.5%) than in the non-edaravone group (11.8%). Moreover, in another randomized study, edaravone suppressed the reduction of serum metalloproteinase 9 levels in 63 patients with acute ischemic stroke [24].

Based upon the efficacy profile of edaravone in patients with multiple stroke subtypes and severities, its potential utility as a neuroprotective treatment should be examined [25]. To achieve that, edaravone should be further studied in a well-controlled clinical study with standardization of dose, treatment duration and time to treatment in order to unequivocally ascertain its efficacy. A recommended time to treatment window in a double-blind randomized clinical trial would be in the range of 7–10 hours based on efficacy trends in patients with various types of stroke [20] and findings in a preclinical embolic stroke translational study [6,26]. The goal of the initial trial should be to provide evidence of significant efficacy using an optimal drug dose within a reasonable therapeutic window, and not obscure efficacy by using an overly broad therapeutic window [25].

### 2.2. Antibiotics

The neuroprotective properties of tetracyclines have been clearly established in rodent models of acute and chronic neurodegeneration during the past few years [27]. Recent findings have provided novel insights into the molecular and cellular mechanisms of protection of neurons and oligodendrocytes by tetracyclines [27].

#### Minocycline

Minocycline is a highly lipophilic semi-synthetic derivative of tetracycline that is a capable of crossing the BBB. Minocycline exerts very promising neuroprotective effects such as inhibition of microglial activity, glutamate toxicity and caspase 1-dependent apoptosis produced by ischemic insults [28–31]. Minocycline attenuates both oxygen-glucose deprivation (OGD)-induced HMGB1 release and HMGB1-induced cell death in PC12 cells subjected to ischemia [32]. In a murine model of middle cerebral artery occlusion, minocycline prevented activation of microglia, which express HMGB1, in the brain, and increased HMGB1 levels in both brain and plasma [33].

One study showed that a significantly good outcome with minocycline treatment could be achieved compared with placebo in patients with acute ischemic stroke [34]. In this open-label, evaluator-blinded study, minocycline at a dose of 200 mg was administered orally for 5 days starting within 6–24 hours after the onset of stroke. The outcome measures were scores on the National Institute of Health Stroke Scale (NIHSS), mRS and BI. The primary objective was to compare changes in NIHSS from baseline to day 90 in the minocycline group *vs.* placebo. NIHSS and mRS scores were significantly lower and BI scores were significantly higher in minocycline-treated patients than placebo-treated patients during days 7–90 of the study. The results of this study suggest that minocycline may have benefits in terms of outcomes (functional recovery outcome) in patients with acute ischemic stroke. Furthermore, a recent study showed that minocycline is safe and well tolerated at doses of up to 10 mg/kg, intravenously, alone and in combination with tPA, in patients with acute ischemic stroke [35]. Despite minocycline being effective in ischemic stroke patients, the small sample size and open-label study indicate the need for a large randomized control trial.

Because the mechanisms of action of edaravone and minocycline are different, combination therapy using these two drugs may be very useful for treating acute ischemic stroke. Such a combination therapy should be further studied to unequivocally ascertain its efficacy in large-scale clinical studies.

### 2.3. Growth Factors

Data from clinical studies provide evidence that growth factors can improve stroke outcome by reducing stroke damage through their anti-apoptotic and anti-inflammatory effects, and by promoting angiogenesis and neurogenesis [36].

#### 2.3.1. Epoetin Beta

Erythropoietin (EPO) has been shown to enhance erythropoiesis under anemic conditions, to have anti-apoptotic and anti-inflammatory effects, to mobilize endothelial progenitor cells into the circulation, and to enhance angiogenesis [37]. Yip *et al.* reported that EPO therapy using epoetin beta significantly reduced the occurrence of major adverse neurological events (defined as recurrent stroke, NIHSS ≥ 8, or death) after treatment for 90 days, compared with placebo, in 167 patients after acute ischemic stroke [38].

#### 2.3.2. Trafermin

Basic fibroblast growth factor (bFGF) has been shown to reduce infarct size in acute ischemic stroke models, and to promote functional recovery as well as new synapse formation when given to animals with completed ischemic stroke [39]. However, a phase II/III randomized trial in 286 patients with acute ischemic stroke confirmed that neither intravenous administration of bFGF, namely, trafermin, nor placebo intravenously infused over 24 hours produced any significant neuroprotection, although intravenous infusion of trafermin did produce dose-dependent hypotension and increased mortality [40].

#### 2.3.3. Filgrastim

In animal models of acute ischemic stroke, granulocyte colony stimulating factor (G-CSF) reduced infarct size, prevented BBB damage, and had an anti-inflammatory effect [36]. However, a phase II, randomized, double-blind trial, namely, AX200 for the treatment of ischemic stroke (AXIS)-2, executed in about 80 renowned stroke centers in Europe, failed to show an effect on clinical outcomes in stroke patients [41]. This study included 328 patients with acute ischemic stroke, one half of which was treated with G-CSF, namely filgrastim, and the other half of which was treated with placebo. Patients were enrolled up to 9 hours after suffering a stroke and were treated by infusion for a period of three days. Patients who had received drug-based lysis therapy using rt-PA prior to treatment with filgrastim were also included in the AXIS-2 Study. No difference in mRS score was observed after 90 days of treatment between the filgrastim and placebo groups [42].

## 3. Prevention of Stroke

### 3.1. Antihypertensive Drugs

Because hypertension, inducing atherosclerosis, is an independent risk factor for stroke [43], antihypertensive treatment is recommended for the prevention of stroke. The preventive effect of antihypertensive drugs against stroke has been researched in many studies, as described below.

#### 3.1.1. Valsartan

The detailed mechanism of action of valsartan [an angiotensin II receptor blocker (ARB)] in the prevention of stroke was confirmed in an animal model. Stroke-prone spontaneously hypertensive rats (SHRSPs), developed from normotensive Wistar Kyoto rats [44], have proven useful for the study of the pathogenesis of stroke and for the testing of prophylactic anti-stroke compounds [45]. SHRSPs develop severe hypertension with age and die from ischemic stroke or hemorrhagic stroke in >80% of the animals [44]. Also, it has been confirmed that, during the generation of spontaneously hypertensive rats (SHRs) and SHRSPs, there is a reduction of cerebral blood flow (CBF) [46,47]. Thus, the etiology of stroke in these rats is very similar to that in humans [44,48]. Valsartan effectively reduced the incidence of stroke in comparison with application of hydralazine in salt-loaded SHRSPs. Despite comparable hypotensive effects between valsartan and hydralazine in salt-loaded SHRSPs, valsartan reduced cerebral NADPH oxidase activity and levels of reactive oxygen species, whereas hydralazine induced only a small amount of reduction in those parameters [49]. Valsartan, but not hydralazine, prevented neuronal apoptosis, and this was associated with the suppression of apoptosis signal-regulating kinase 1 activation by valsartan. Moreover, cerebral inflammation was also prevented by valsartan, and this was associated with suppression of inflammatory cytokines such as monocyte chemoattractant protein1 (MCP1) and tumor necrosis factor (TNF)-α by valsartan.

The results of two recent intervention trials, namely, the Japanese Investigation of Kinetic Evaluation in Hypertensive Event and Remodeling Treatment (JIKEI HEART) study and the Kyoto Heart study, both conducted in Japan, have suggested a neuroprotective effect of valsartan against stroke events [50,51]. The JIKEI HEART study was a multicenter, prospective, randomized controlled trial of 3081 patients who were undergoing conventional treatment for hypertension, coronary heart disease, heart failure, or a combination of these disorders [50]. In addition to conventional treatment, patients were assigned either to valsartan (ARB) or to other treatments without ARB. New or recurrent stroke was significantly reduced, by 40%, in the valsartan group. The addition of valsartan to conventional treatment prevented more stroke events than did supplementary conventional treatment.

The Kyoto Heart study was a multicenter study with a PROBE design [51]. A total of 3031 patients with uncontrolled hypertension were randomized to either valsartan add-on or non-ARB treatment. Valsartan add-on treatment prevented more stroke events than did conventional non-ARB treatment in high-risk hypertensive patients in Japan. Valsaratan significantly reduced the incidence of stroke events, by 45% in this study. The significant reduction in the number of stroke events in the Kyoto Heart study was consistent with that reported in the JIKEI HEART study.

#### 3.1.2. Losartan, Eprosartan and Telmisartan

The effects of other ARBs, namely, losartan, eprosartan and telmisartan, against stroke, were also studied. Losartan reduced systolic blood pressure in salt-loaded SHRs. Losartan also significantly attenuated hypertension-related vascular remodeling, aortic malondialdehyde accumulation, lowering of renal artery endothelial nitric oxide synthase activity, and reductions in aortic and renal artery superoxide dismutase activity [52]. The Losartan Intervention For Endpoint reduction in hypertension (LIFE) study compared losartan and the β-blocker atenolol in 9193 hypertensive patients with electrocardiographically demonstrated left ventricular hypertrophy [53]. Losartan significantly reduced the incidence of stroke, by 25% compared with atenolol in this study.

Eprosartan is an ARB that inhibits the hypertensive effects of AT II and norepinephrine by competitively blocking AT II type 1 (AT1) receptors in the circulation and on sympathetic neurons. This unique dual mechanism of action may confer benefits over other antihypertensive agents in terms of greater reductions in the incidence of strokes [54]. Few clinical trials have directly compared different classes of blood pressure-lowering drugs after TIA or stroke [55]. In the Morbidity and Mortality After Stroke, Eprosartan Compared with Nitrendipine for Secondary Prevention (MOSES) study, 1450 hypertensive patients with a stroke or TIA within the previous 2 years were randomly assigned to receive eprosartan or nitrendipine (a calcium-channel blocker) [56]. The amounts of reduction in blood pressure produced by the two agents were similar, but the risk of stroke or TIA was lower with eprosartan. There was a significant reduction of 25% in the recurrence of stroke and associated diseases such as transient ischemic attack (TIA) in those treated with eprosartan.

Telmisartan is a unique AT1 receptor blocker with a peroxisome proliferator-activated receptor gamma (PPAR-γ) agonistic action [57]. Activation of PPAR-γ could prevent inflammation and brain damage. Furthermore, telmisartan was more effective than losartan in increasing CBF, and in reducing TNF-α and MCP1 expression levels in diabetic mice [57]. The recently published Ongoing Telmisartan Alone and in Combination with Ramipril Global Endpoint Trial (ONTARGET) study compared ramipril (an ACE inhibitor), telmisartan and a combination of both drugs in 25,611 patients with vascular disease or high-risk diabetes mellitus (DM) over a median follow-up period of 56 months [58]. Ramipril, telmisartan and combination therapy proved to be equivalent with regard to the prevalence of recurrent stroke. However, the Prevention Regimen For Effectively avoiding Second Strokes (PRoFESS) study failed to show a benefit for telmisartan in reducing the risk of recurrent stroke compared with placebo [59]. The results of this study showed that telmisartan is no better than any other antihypertensive drug for the prevention of a second stroke.

Valsartan reduced the incidence of stroke events by 40–45%, whereas losartan and eprosartan reduced the incidence of stroke events by 25%. However, the control condition used in each study was different. Therefore, a comparison of the reduction in incidence of stroke events may not be suitable in a discussion of the effectiveness of these drugs. At the very least, however, it is clear that ARBs can reduce the incidence of stroke events. It may be difficult to simultaneously compare the effects of these ARBs in terms of their reduction of stroke incidence in a clinical study; therefore, a study using SHRSPs to compare the effects of these ARBs may be the most plausible means to compare of the neuroprotective effects of each ARB against stroke.

#### 3.1.3. Perindopril

A meta-analysis revealed that ARBs significantly reduced the incidence of stroke by just 8% when compared with angiotensin-converting enzyme (ACE) inhibitors [60]. Meanwhile, the beneficial effect of perindopril (ACE inhibitor) may be associated with CBF. Long-term therapy with perindopril improved cerebral circulation in patients with previous minor stroke [61]. Thybo *et al.* reported that perindopril treatment for 12 months resulted in a more prominent increase in small artery diameter and a greater reduction in the media-lumen ratio than did treatment with the β-blocker atenolol [62]. The results of the Perindopril Protection Against Recurrent Stroke Study (PROGRESS) showed that 6105 patients with a prior stroke or TIA who received perindopril-based therapy significantly decreased the risk of secondary stroke, by 28% *versus* placebo [63].

### 3.2. Lipid-Lowering Drugs

Because hypercholesterolemia, inducing atherosclerosis, is a major risk factor for stroke [64,65], treatment with statins (HMG-CoA reductase inhibitors) and ω-3 fatty acids is recommended for the prevention of stroke [66,67]. The preventive effects of such treatments against stroke have been researched in many studies as described below.

#### 3.2.1. Atorvastatin

Several lines of evidence have shown that atorvastatin has diverse effects. Short-term and low-dose atorvastatin therapy was given to patients with chronic cerebrovascular disease and hyperlipidemia, revealing the following effects of atorvastatin: lowered lipid levels; decreased collagen-induced platelet aggregation; improved whole blood viscosity; improved red blood cell deformability; improved von Willebrand factor activity; and improved endothelial dysfunction [68]. Furthermore, atorvastatin decreased markers of oxidative stress in hypercholesterolemic patients [69], and inhibited inflammatory angiogenesis in mice through down-regulation of vascular endothelial growth factor, TNF-α and transforming growth factor β1 [70].

The Myocardial Ischemia Reduction with Aggressive Cholesterol Lowering (MIRACL) study enrolled 3086 patients hospitalized for acute coronary syndromes [71]. Subjects were randomized immediately after admission to receive atorvastatin or placebo for 16 weeks. The relative risk (RR) of stroke in the atorvastatin group compared with the placebo group was significantly reduced, by 51%.

In the Greek Atorvastatin & Coronary Heart Disease Evaluation (GREACE) study, 1600 patients with established CHD and LDL levels >100 mg/dl were randomized to treatment with atorvastatin or with usual care [72]. After 3 years of follow-up, the incidence of stroke was significantly lowered, by 47%.

The Anglo-Scandinavian Cardiac Outcomes Trial (ASCOT) study included hypertensive patients who also had risk factors for cardiovascular disease [73]. A total of 10,305 patients with non-fasting total cholesterol concentrations <6.5 mmol/L were randomly assigned to receive additional atorvastatin or placebo. The incidence of stroke was also significantly reduced by atorvastatin in this study, this time by 27%.

In the Collaborative Atorvastatin Diabetes Study (CARDS), 2,838 patients were randomized to receive atorvastatin or placebo [74]. Participants had no documented previous history of cardiovascular disease, an LDL-cholesterol concentration of 4.14 mmol/L or lower, a fasting triglyceride level of 6.78 mmol/L or less, and at least one of the following: retinopathy, albuminuria, current smoking or hypertension. The incidence of stroke was also significantly reduced by 48% with atorvastatin.

The Stroke Prevention by Aggressive Reduction in Cholesterol Levels (SPARCL) study was the first study to evaluate the role of statins in secondary prevention of stroke [75]. Overall, 4731 patients who had experienced a TIA or minor stroke within the previous 6 months and a level of low-density lipoprotein-cholesterol of 100–190 mg/dl were randomized to receive a high dose of atorvastatin or placebo. After a mean period of 4.9 years, there was a 16% RR reduction for subsequent stroke in patients treated with atorvastatin, and this reduction was significant.

The complex beneficial effects of atorvastatin may prevent stroke. Among the various clinical studies of agents for the prevention of stroke, atorvastatin may be the most effective statin. However, no significant reduction in the incidence of stroke was found in some clinical studies of atorvastatin, such as the Aggressive Lipid-Lowering Initiation Abates New Cardiac Events (ALLIANCE) and Atorvastatin Study for Prevention of Coronary Heart Disease End points in Non-Insulin-Dependent Diabetes Mellitus (ASPEN) studies [76,77].

#### 3.2.2. Simvastatin

The effect of simvastatin on prevention of stroke was confirmed in an animal model. Simvastatin ameliorated cerebrovascular remodeling in the hypertensive rat through inhibition of vascular smooth muscle cell proliferation by suppression of volume-regulated chloride channels [78].

In the Scandinavian Simvastatin Survival Study (4S), 4444 patients with angina pectoris or previous MI and serum cholesterol 5.5–8.0 mmol/L on a lipid-lowering diet were randomized to double-blind treatment with simvastatin or placebo [79]. Over the 5.4 years median follow-up period, simvastatin significantly lowered the incidence of stroke, by 30%.

The Heart Protection Study (HPS) compared simvastatin and placebo in 20,536 patients during a treatment period of 5 years [80]. The patients had coronary artery disease, other occlusive vascular disease (16% of the entire study population had a history of stroke), DM, or arterial hypertension and other risk factors. Although there was a highly significant 25% proportional reduction in the first event rate for stroke, there was no significant reduction in the recurrence of stroke in this study. The discrepancy in the prevalence of recurrent ischemic stroke between the HPS and SPARCL studies may be due to the length of the follow-up period; patients in the HPS study were recruited, on average, 4.3 years after the initial vascular event, whereas this time interval was only 6 months in the SPARCL study [81].

Meanwhile, no significant reductions in the incidence of stroke were found in other clinical studies of simvastatin, such as the Aggrastat to Zocor (A to Z) study and the Study of the Effectiveness of Additional Reduction in Cholesterol & Homocysteine (SEARCH) [82–84].

Furthermore, the Incremental Decrease in End Point Through Aggressive Lipid Lowering (IDEAL) study, a prospective, randomized, open-label, blinded end point evaluation trial with a median follow-up of 4.8 years, enrolled 8888 patients with a history of acute MI [85]. Patients were randomly assigned to receive either atorvastatin (80 mg/day) or simvastatin (20 mg/day). After follow-up, stroke was infrequent but, again, the rates did not differ significantly between the two groups.

#### 3.2.3. Pravastatin

The neuroprotective effect of pravastatin was investigated in an animal model. Insulin resistance is an independent risk factor for stroke [86]. Endothelial dysfunction in response to risk factors and carotid artery disease are important in the pathogenesis of stroke. Pravastatin restored endothelial function in carotid arteries from insulin-resistant rats with fructose-induced hypertension. These restoration effects were ascribed to direct, cholesterol-independent vascular effects of pravastatin, and are likely the result of augmentation of nitric oxide production.

The CARE study of 4159 patients with MI before randomization evaluated the beneficial effects of pravastatin in comparison with placebo. Patients had plasma total cholesterol levels below 240 mg/dl and low-density lipoprotein (LDL) cholesterol levels of 115–174 mg/dL [87]. Pravastatin significantly reduced the rate of stroke by 31% compared with placebo.

The Long-Term Intervention with Pravastatin in Ischemic Disease (LIPID) study of 9014 randomized patients with a history of MI or hospitalized for unstable angina and initial plasma total cholesterol levels of 155–271 mg/dL, was conducted to evaluate treatment with pravastatin *versus* placebo [88]. Pravastatin significantly reduced the rate of stroke by 19% compared with placebo [88,89].

In the Management of Elevated Cholesterol in the Primary Prevention Group of Adult Japanese (MEGA) prospective, randomized, open-label, blinded study, Japanese 7832 patients with hypercholesterolemia and no history of coronary heart disease (CHD) or stroke were randomly assigned to begin a diet or a diet plus pravastatin [90]. At 5 years, significant reductions in the incidence of stroke by 32% were noted. Treatment with a low dose of pravastatin reduces the risk of stroke in Japan by much the same amount as higher doses have been shown to do in Europe and the USA.

Meanwhile, there were no significant reductions in the incidence of stroke in some clinical studies such as the West of Scotland Coronary Prevention Study (WOSCOPS), the Kyushu Lipid Interventional Study (KLIS), the Gruppo Italiano per lo Studio della Sopravvivenza nell’ Infarto del Miocardio (GISSI) study, the Pravastatin in Elderly Individuals at Risk of Vascular Disease (PROSPER) study and the Antihypertensive and Lipid-Lowering Treatment to Prevent Heart Attack Trial (ALLHAT-LLT) [91–95].

The Thrombolysis In Myocardial Infarction (TIMI)-22 study enrolled 4162 patients who had been hospitalized for acute coronary syndrome within the preceding 10 days, and compared atorvastatin 80 mg/day with pravastatin 40 mg/day [96]. After a mean follow-up period of 24 months, stroke was infrequent but the rates did not differ significantly between the two groups.

#### 3.2.4. Rosuvastatin

The protective effect of rosuvastatin was compared with that of simvastatin in an animal model. Rosuvastatin attenuated inflammatory processes associated with cerebrovascular disease. In comparison with simvastatin-treated SHRSPs, rosuvastatin treatment attenuated the transcription of inflammatory mediators such as MCP1, interleukin 1β and TNF-α in the kidney, and that of P-selectin in brain vessels, while increasing the transcription of endothelial nitric oxide synthase mRNA in the aorta [97].

In the Justification for the Use of Statins in Prevention: an Interventional Trial Evaluating Rosuvastatin (JUPITER) study, 17,802 apparently healthy men and women were randomly assigned to rosuvastatin or placebo [98]. Patients with normal lipid levels but elevated high-sensitivity C-reactive protein showed a 48% reduction in the risk of stroke when taking rosuvastatin, a reduction that was significant.

Although rosuvastatin is a strong statin like atorvastatin, there have been few clinical studies of this drug for stroke prevention. Atorvastatin has most useful evidence for a possible stroke preventive effect compared with other statins. Atorvastatin may be the most effective statin; however, no significant reductions in the incidence of stroke were found in two clinical studies when the use of atorvastatin was compared with other statins, such as simvastatin and pravastatin. These two clinical studies were the IDEAL and TIMI-22 studies. Atorvastatin and simvastatin are lipophilic; therefore, both are able to cross the BBB. By contrast, pravastatin is hydrophilic, and is not able to cross the BBB [99]. Brain cholesterol may not influence the incidence of stroke directly. Vaughan *et al.* hypothesized that the stroke prevention effect of statins could be attributed to their cholesterol-independent (pleiotropic) effects. These include antithrombotic, antioxidative, anti-inflammatory, vasodilatory, and plaque-stabilizing mechanisms that favorably affect atherosclerosis and mediate neuroprotection indirectly [100].

#### 3.2.5. Eicosapentaenoic Acid

The mechanism of action underlying the neuroprotective effect of eicosapentaenoic acid (EPA) was investigated in an animal model. Katayama *et al.* reported that the effect of EPA on prevention for stroke may be due to the amelioration of CBF and glucose utilization in SHRSPs [45].

The Japan Eicosapentaenoic acid Lipid Intervention Study (JELIS) examined the preventive effect of EPA against the first and recurrent stroke [101]. EPA is contained in fish oil and is given at a dose of 1800 mg/day using 300-mg capsules of highly purified (>98%) EPA ethyl ester. Hypercholesterolemic patients received statin only or statin with EPA for around 5 years. No statistically significant difference in total stroke incidence was observed between the two groups. The study showed that the incidence of recurrent stroke was reduced by 20% in the EPA group. Administration of highly purified EPA appeared to reduce the risk of recurrent stroke in a Japanese population of hypercholesterolemic patients receiving low-dose statin therapy. Further research is needed to determine whether similar benefits occur in other populations with lower levels of fish intake.

### 3.3. Hypoglycemic Drugs

Because DM-induced atherosclerosis increases the risk of stroke [102], glycemic control is recommended for the prevention of stroke. The preventive effect of glycemic control for stroke has been researched in only one study, described below.

#### Pioglitazone

Pioglitazone has diverse effects: it inhibits oxidative stress such as by reducing serum nitrotyrosine levels in patients with type-2 diabetes mellitus (T2DM) [103]; it increases adiponectin levels in patients with metabolic syndrome [104]; and it improves endothelial dysfunction in cerebral vessels in patients with T2DM [105]. The combined effects of pioglitazone other than its hypoglycemic effects may prevent secondary stroke. Pioglitazone is an orally administered insulin-sensitizing thiazolidinedione agent that has been developed for the treatment of T2DM [106]. Recurrent stroke was significantly reduced by 47% in patients with a prior history of stroke who received pioglitazone compared with those treated with placebo in the Prospective Pioglitazone Clinical Trial in Macrovascular Events (PROACTIVE) study [107].

Recently, some preclinical in vivo studies and limited human data have suggested a possible increased risk of bladder cancer with pioglitazone therapy [108]. Short-term use of pioglitazone was not associated with an increased incidence of bladder cancer, but use for more than 2 years was weakly associated with increased risk in another cohort study of 193,099 patients with diabetes [109]. Furthermore, in the results of a cohort study of 207,714 patients with T2DM suggest that pioglitazone may not be significantly associated with an increased risk of bladder cancer in patients with T2DM [110]. Thus, this side effect of pioglitazone is not clear.

Because few clinical studies on the prevention of stroke have evaluated other glycemic control drugs, pioglitazone may be the most effective drug for the treatment of stroke among currently available drugs for hypoglycemia. More clinical studies of other glycemic control drugs are expected in the future.

## 4. Conclusions

Both treatment and prevention of stroke are crucial issues given that stroke is a frequent and severe disorder, and stroke therapies (which are effective at the individual level) have only a limited impact on public health (Table 1). To prevent stroke, we should focus on reducing vascular risk factors such as high blood pressure, high blood cholesterol, and high blood glucose. To reduce the risk of vascular events, antihypertensive drugs, lipid-lowering drugs, and hypoglycemic drugs may be candidates for providing good outcomes. A complementary strategy is the optimal management of the risk factors for stroke; therefore, choosing the optimal oral drugs for the prevention of stroke is important. Furthermore, various drugs other than tPAs, antiplatelet agents and anticoagulants should be initiated urgently when stroke occurs. It has been hypothesized that no single drug will have maximal efficacy and that a cocktail approach may be needed to promote neuroprotection [111]. Thus, success may come with methods that enable screening of combination therapies. A combination therapy strategy may provide synergy of effects. It is even likely that multiple pleiotropic drugs will be combined to treat stroke [112]. Cocktail therapy with a combination of edaravone and minocycline in addition to antithrombotic drugs may be the core component of the optimal therapy for acute stroke. There is insufficient evidence to support stroke treatment using growth factors; however, combination therapy with epoetin beta and tPA should be performed in a clinical study. Meanwhile, it is not easy to select optimal ARB and statin combinations for the prevention of stroke, because there are few clinical studies in which drug combinations have been compared directly with regard to their effects on the prevention of stroke. Randomized, controlled clinical studies of cocktail therapies with large sample sizes should be performed. We should remain optimistic about the future of therapy development for stroke and continue to explore new scientific strategies to provide optimal care to stroke patients.

## Figures and Tables

**Table 1:** Beneficial neuroprotective effects of the various clinical drugs in clinical studies.

Drugs	Original effect	Effect for stroke	Sample size	Reference
Edaravone	Free radical scavenger	Acute treatment	125	[22]
Edaravone	Free radical scavenger	Acute treatment	72	[24]
Edaravone	Free radical scavenger	Acute treatment	41	[23]
Edaravone	Free radical scavenger	Acute treatment	401	[25]
Edaravone	Free radical scavenger	Acute treatment	40	[21]
Edaravone	Free radical scavenger	Acute treatment	63	[26]
Minocycline	Antibiotics	Acute treatment	152	[36]
Epoetin beta	Growth factor	Acute treatment	167	[40]
Valsartan	Antihypertensive	Primary prevention	3,081	[50]
Valsartan	Antihypertensive	Primary prevention	3,031	[51]
Losartan	Antihypertensive	Primary prevention	9,193	[53]
Eprosartan	Antihypertensive	Secondary prevention	1,450	[56]
Telmisartan	Antihypertensive	Secondary prevention	2,5611	[58]
Perindopril	Antihypertensive	Secondary prevention	6,105	[62]
Atorvastatin	Lipid lowering	Primary prevention	3,086	[71]
Atorvastatin	Lipid lowering	Primary prevention	1,600	[72]
Atorvastatin	Lipid lowering	Primary prevention	10,305	[73]
Atorvastatin	Lipid lowering	Primary prevention	2,838	[74]
Atorvastatin	Lipid lowering	Secondary prevention	4,731	[75]
Simvastatin	Lipid lowering	Primary prevention	4,444	[79]
Simvastatin	Lipid lowering	Primary & Secondary prevention	20,536	[80]
Pravastatin	Lipid lowering	Primary prevention	4,159	[87]
Pravastatin	Lipid lowering	Primary prevention	9,014	[89]
Pravastatin	Lipid lowering	Primary prevention	7,832	[90]
Rosuvastatin	Lipid lowering	Primary prevention	17,802	[98]
Eicosapentaenoic acid	Lipid lowering	Secondary prevention	942	[101]
Pioglitazone	Glycemic control	Secondary prevention	948	[107]

## List of Abbreviations

4S: Scandinavian Simvastatin Survival Study
ACE: Angiotensin-converting enzyme
ALLHAT-LLT: Antihypertensive and Lipid-Lowering Treatment to Prevent Heart Attack Trial
ALLIANCE: Aggressive Lipid-Lowering Initiation Abates New Cardiac Events
AQP4: Aquaporin4
ARB: Angiotensin II receptor blocker
ASCOT: Anglo-Scandinavian Cardiac Outcomes Trial
ASPEN: Atorvastatin Study for Prevention of Coronary Heart Disease End points in Non-Insulin-Dependent Diabetes Mellitus
AT1: Angiotensin II receptor type 1
A to Z: Aggrastat to Zocor
AXIS: AX200 for the treatment of ischemic stroke
BBB: Blood–brain barrier
bFGF: Basic fibroblast growth factor
BI: Barthel Index
CARDS: Collaborative Atorvastatin Diabetes Study
CARE: Cholesterol and Recurrent Events
CBF: Cerebral blood flow
CHD: Coronary heart disease
DM: Diabetes mellitus
EPA: Eicosapentaenoic acid
EPO: Erythropoietin
FIM-M: Functional independence measure-motor
G-CSF: granulocyte colony stimulating factor
GISSI: Gruppo Italiano per lo Studio della Sopravvivenza nell’ Infarto del Miocardio
GREACE: Greek Atorvastatin & Coronary Heart Disease Evaluation
HMGB1: High-mobility group box 1
HPS: Heart Protection Study
IDEAL: Incremental Decrease in End Point Through Aggressive Lipid Lowering
JELIS: Japan Eicosapentaenoic acid Lipid Intervention Study
JIKEI HEART: Japanese Investigation of Kinetic Evaluation in Hypertensive Event and Remodeling Treatment
JUPITER: Justification for the Use of Statins in Prevention an Interventional Trial Evaluating Rosuvastatin
KLIS: Kyushu Lipid Interventional Study
LDL: Low-density lipoprotein
LIFE: Losartan Intervention for Endpoint Reduction in Hypertension
LIPID: Long-Term Intervention with Pravastatin in Ischemic Disease
MCP1: Monocyte chemoattractant protein 1
MEGA: Management of Elevated Cholesterol in the Primary Prevention Group of Adult Japanese
MI: Myocardial infarction
MIRACL: Myocardial Ischemia Reduction with Aggressive Cholesterol Lowering
MOSES: Morbidity and Mortality after Stroke, Eprosartan Compared with Nitrendipine for Secondary Prevention
mRS: Modified Rankin scale
NIHSS: National Institute of Health Stroke Scale
OGD: Oxygen-glucose deprivation
ONTARGET: Ongoing Telmisartan Alone and in Combination with Ramipril Global Endpoint Trial
PROBE: Prospective Randomized Open Blinded Endpoint
PROSPER: Pravastatin in Elderly Individuals at Risk of Vascular Disease
RR: Relative risk
PROACTIVE: Prospective Pioglitazone Clinical Trial in Macrovascular Events
PRoFESS: Prevention regimen For Effectively avoiding Second Strokes
PROGRESS: Perindopril Protection Against Recurrent Stroke Study
SEARCH: Study of the Effectiveness of Additional Reduction in Cholesterol & Homocysteine;
SHR: Spontaneously hypertensive rats
SHRSP: Stroke-prone spontaneously hypertensive rats
SPARCL: Stroke Prevention by Aggressive Reduction in Cholesterol Levels
T2DM: Type-2 diabetes mellitus
TIA: Transient ischemic attack
TIMI: Thrombolysis in myocardial infarction
TNF: Tumor necrosis factor
tPA: Tissue plasminogen activator
WOSCOPS: West of Scotland Coronary Prevention Study
